# Nodal Status Assessment in Breast Cancer: Strategies of Clinical Grounds and Quality of Life Implications

**DOI:** 10.1155/2014/469803

**Published:** 2014-02-11

**Authors:** Paolo Orsaria, Dimitrios Varvaras, Gianluca Vanni, Giampiero Pagnani, Jacopo Scaggiante, Federico Frusone, Alessandra Vittoria Granai, Giuseppe Petrella, Oreste Claudio Buonomo

**Affiliations:** Department of Surgery, Tor Vergata University Hospital, 00133 Rome, Italy

## Abstract

Even in the era of gene-expression profiling, the nodal status still remains the primary prognostic discriminant in breast cancer patients. The exclusion of node involvement using noninvasive methods could reduce the rate of axillary surgery, thereby preventing from suffering complications. However, lymphatic mapping with sentinel node biopsy (SNB) is one of the most interesting recent developments in surgical oncology. Optimization of procedure could be implemented by dual mapping injection site skills, resection of all hot or blue nodes through tracer combination, and improvement in atypical drainage patterns mapping. This anatomical analysis suggests safety measures in patients with high probability of node metastasis through a renewed interest in surgical management. The perspective of a guided axillary sampling (GAS) could represent a potential development of recent anatomical and functional acquisitions, offering a dynamic technique shared according to clinical and anatomical disease parameters. Furthermore, the surgical staging procedures may adopt a conservative approach through the evaluation of upper arm lymphatics, thus defining a functional model aimed at the reduction of short- and long-term adverse events. Quality results in breast cancer surgery need to generate oncological safety devoid of complications through renewed clinical experience.

## 1. Introduction

### 1.1. Health Problem: Breast Cancer and Axillary Metastases

Overall, breast cancer five-year age-standardized survival rates are around 80%. Survival varies with age and stage of disease from 88–69% (I-II) to 43–12% (III-IV) [1]. Although newer markers of oncogene expression show promise with respect to treatment of disease, the nodal status still remains the primary prognostic discriminant and is important for tailoring treatment. Axillary nodes are the most common sites of expansion outside the breast, occurring in approximately 41% of cases, and prognosis is better when there is no lymphatic invasion [2]. Additional large, well-conducted studies are required to obtain more accurate data on sensitivity and specificity of imaging techniques, in addition to the accuracy and costs of the different diagnostic methods. Where metastases are present, surgical removal of axillary nodes is indicated in order to ensure staging accuracy and local disease control. Furthermore, even a combination modality of three noninvasive diagnostic imaging techniques (US, PET-TC, and MRI) cannot substitute for an invasive method to make decisions for appropriate systemic interventions. Traditional staging requires dissection in Level I and II axillary lymph nodes (ALND) with 10 or more removed nodes. ALND is very accurate in establishing the presence of axillary disease and has the therapeutic advantage of being associated with a high long-term local disease control rate. However, ALND is associated with significant complications (e.g., 21% of arm lymphedema, 22% of seromas, and 14% of infection rate) [3]. In recent years, however, sentinel lymph node biopsy (SLNB) has been proven to be a feasible, accurate, and suitable method for staging the axilla, while avoiding the morbidity of ALND, in patients with clinically node-negative breast cancer on clinical examination, ultrasound, or fine needle aspiration cytology [4]. As a minimally invasive approach, SLNB has become a standard surgical technique in the management of early breast cancer patients whereby prevalence is assumed fixed at 41.2% [5]. A reduction in morbidity is an obvious goal but the more challenging metric is demonstrating that survival is not adversely affected. Sentinel node surgery represents the next major step in reducing the extent of surgical procedures, but, despite the revolution of quality conserving care, recent data collection and analyses using anatomical techniques suggest that the exact lymphatic drainage of the breast continues to be debatable [6]. Different lymphatic patterns may help to explain some important unresolved clinical problems, including different detection rates in different studies and high false-negative rates of about 10% in multicenter randomized controlled trials [7]. Further anatomical investigation and knowledge of the exact sentinel lymphatic channels (SLCs) will provide more underlying information about patterns of breast cancer in order to improve surgical strategy, locoregional recurrence, and survival.

## 2. Diagnostic Accuracy and Implication for Service Provision

### 2.1. Clinical Results of Imaging Diagnostic Pathway

In the era of SLNB, the exclusion of nodal involvement using noninvasive methods could reduce the rate of axillary surgery, thereby preventing patients without lymph node (LN) metastases from suffering complications. Therefore, the sensitivity, specificity, and accuracy of diagnostic imaging techniques (US, PET-TC, and MRI) have been appropriately examined (Table 1). Ultrasound (US) is currently recommended prior to surgical assessment of the axilla for all patients with early-stage breast cancer. A systematic review estimated the average sensitivity at 44–61% in patients with nonpalpable axillary nodes and the specificity at 75–86% in all patients. Furthermore, if clinical and US scans suggest nodal metastases on the basis of size or abnormal morphology, US-guided biopsy (FNAC or Core biopsy) of abnormal nodes is undertaken, which detects 45% of metastases [8]. Preoperative axillary US is a widely accepted diagnostic method that provides additional value in detecting pathological spread but on its own is insufficiently specific to obviate the need for SLNB because of the substantial number of FN results, particularly in N1 disease, although it may almost exclude N2 and N3 disease [9]. A large number of prospective randomized studies must be completed for validation of performance results with additional relevant costs. Positron emission tomography (PET-TC) is a nuclear medicine imaging technique that produces a three-dimensional map of functional processes in the body. Across 26 studies (*n* = 2591 patients) evaluating PET or PET-CT or PET only for assessment of axillary metastases, the mean sensitivity was 56–66% and the mean specificity was 93–96%. However, there was a trend for lower sensitivities where metastatic lymph nodes were smaller or less in number. Micrometastases (>2 mm) were associated with a mean sensitivity of 11% based on data from five studies (*n* = 63), while macrometastases (>2 mm) were associated with a mean sensitivity of 57% based on data from four studies (*n* = 111) [5]. A further meta-analysis of 25 studies including 2460 patients reported that PET-CT provided lower sensitivity (37% to 85%) and high specificity (84% to 100%). Compared with the combination of breast sonography and mammography, PET-CT was less sensitive and had less accuracy in detecting node disease. Consequently, it is not a reliable noninvasive modality to assess node involvement to replace ALND or SLNB before decisions are made on appropriate systemic interventions [10]. MRI scanning may provide information on whether a lesion is suspicious for metastasis based on criteria such as size, morphology, and enhancement characteristics following administration of a contrast agent. Several MRI studies reported more than one set of diagnostic accuracy results, according to different criteria, but the contrast of use and uptake pattern was the main requirement for defining a node as metastatic with a better combined sensitivity (90%) and specificity (90%) [11, 12]. The morphological features of lymph node enhancement defects and dilated lymphatic vessels show potential for differentiating metastatic nodes through magnetic resonance lymphangiography (MRL). The physiology of MRL makes the identification of SLNs readily apparent due to gadolinium-based contrast uptake within lymphatic vessels and lymph nodes following intradermal injection, but further investigations would have to be performed to identify the accuracy of new biomarkers on these pathological findings [13]. In order to evaluate the effects on patient outcomes and cost-effectiveness of enhanced imaging techniques compared with the standard assessment, Cooper et al. modified the standard pathway. Key findings from diagnostic results suggested that the most cost-effective strategy might be MRI or PET rather than SLNB or 4-NS, reducing costs, and increasing quality of life years due to fewer adverse events for the majority of patients. However, the two strategies of replacing axillary sampling with imaging techniques may be considered unacceptable on clinical grounds due to the higher numbers of FN cases (leading to higher risk of recurrence) and FP cases (leading to unnecessary ALND). In the MRI replacement strategy, the number of FP cases increased significantly from 0.2% to 6.3% and the number of FN cases increased to a lesser extent, from around 1.0% to 1.9%. In the PET replacement strategy the numbers of both FP and FN cases increased significantly, from 0.2 to 3.6% for FP cases and from around 1.0% to 7.2% for FN cases [5]. Positron emission tomography and MRI are assumed to be associated with neither short nor long term adverse events. However, due to the lower accuracy of the imaging techniques, more FP and FN cases will be produced, which will lead to increased costs, poorer quality of life due to adverse events, and, in some cases, a higher probability of recurrence and subsequent death from breast cancer. Kwak's et al. evaluation of SLNB in 323 patients with breast cancer suggests that no imaging techniques like US, MRI, and PET-CT can replace surgical staging and histologic confirmation of nodal status. The presence of axillary LN metastasis on preoperative imaging carried 82.1% sensitivity, 45.9% specificity, 33.8% positive predictive value, and 86.1% negative predictive value for determining axillary metastasis on final pathology [14]. The aim of this data review was also to determine whether it is safe and feasible to perform SLNB in patients with clinically suspicious axillary nodes in preoperative imaging studies, which showed that the sentinel procedure works well in a wide range of practice settings. Furthermore, alternative imaging addition diagnostic pathway demonstrated that the sensitivity and specificity of both PET and MRI before sampling methods vary significantly between studies. The advantage of adding strategy before sampling results in fewer FN cases (reduced from around 1.0% to 0.1% for MRI and to around 0.5% if PET is placed before sampling) due to the use of two sequential tests and fewer sampling procedures performed because sampling methods are avoided if MRI or PET results are positive. The disadvantages of these strategies are that there are more FP cases because the specificities of MRI and PET are lower than those of SLNB and 4-NS. FP increases from 0.2 to 6.3% for MRI prior to SLNB, which is the same as for the MRI replacement strategy [5]. In conclusion, in order to have similar levels of FP and FN cases for the sampling methods, the specificity of MRI and PET needs to be improved by close to 100%, which by definition is the specificity of 4-NS and SLNB. However, availability of PET and MRI scanning facilities would need to be considered if PET or MRI was recommended as part of the routine screening pathway for all patients with early breast cancer.

### 2.2. The Sentinel Node Procedure in Breast Cancer

The axilla must be explored surgically because imaging techniques have limited sensitivity. Axillary involvement is found in 10–30% of patients with T1, depending on size, reaching 45% for small T2 tumors (2.1–3 cm) and 55–70% for larger tumors [15]. Randomized trials in which the primary aim was to assess morbidity conclusively demonstrated a marked diminution of complications associated with the SLNB strategy when compared with routine ALND. In the ALMANAC outcome measures, the twelve-month risks of lymphedema and sensory loss after surgery were, respectively, 13% and 31% in the ALND group compared to 5% and 11% in the SLNB group [16]. Furthermore, specific results concerning the impact of sentinel strategy on recurrence and survival have confirmed the oncologic safety of healthy lymph nodes preservation. The eligibility criteria for a procedure are the main elements of a prognostic management classification system, but new quality indicators still need to be incorporated into clinical practice to evaluate the applicability and relevance for surgeons. When the authors excluded patients with tumors larger than 2 cm or multicentric, with prior excisional biopsy, younger than 40 years, or when a sentinel node was not found at lymphoscintigraphy and preoperative probe-guided inspection, the axillary relapse rate in the SLNB group was as low as 1.2% per 10-year follow-up [17]. Although this data is fully reassuring, the adopted wide exclusion criteria might limit the generalization of these conclusions in order to develop a more practical approach to quality assessment.

All studies on SLN biopsy, according to established breast cancer quality indicators (QIs), report a variable false-negative rate whose prognostic consequences are still unclear [17–25] (Table 2). At a median follow-up of 56 months, Zavagno et al. reported positive non-SLNs rate of 16.7% and there were more locoregional recurrences in the SLN arm than in patients randomly assigned to receive axillary lymph node dissection [18]. It is highly debated which patients can be offered sentinel surgery but the clinical research could benefit from new operational perspectives since it represents the next major step in reducing the extent of surgical procedures to treat breast cancer. False-negative SLNB results might impair patient outcomes for several reasons since missed nodes might lead to axillary recurrence that is difficult to treat and understaging could affect decisions about systemic and radiation therapy. In Hindié's et al. systematic review, results from large multi-institutional series showed that all have achieved excellent identification rates, ranging from 93.5% to 97.2%, but none achieved an FN rate lower than 5%, with a weighted average of 9.2%. Identification rate may thus serve as a reasonable quality indicator for sentinel biopsy because the lowest FN rates were obtained in the studies in which preoperative lymphoscintigraphy and dual mapping during surgery were required [26]. Dual mapping with radiotracer and blue dye, combining 2 different injection sites, and routinely using lymphoscintigraphy may improve accuracy. By providing the surgeon with a map of sentinel lymphatic channels, the new anatomical and functional acquisitions could validate the resection of all hot or blue spread, thereby showing the influence of the quantity of removed nodes on the FN rate. The different techniques used to identify the sentinel node form the basis for defining a concept which goes beyond any definition: the lymphatic drainage. The SLNB procedure uses radiotracers, dyes, or nanoparticles, and at the same time, produces different results including “the node closest to the primary lesion,” “the first node visualised at lymphoscintigraphy,” “the hottest node,” “all radioactive nodes,” “all blue nodes,” or “all nodes with a count rate that is a certain factor higher than that of the background or compared to other nodes.” The administration of radiopharmaceuticals may result in acceptable accuracy according to the introduction of a threshold of percentual activity in order to distinguish SNs from nSNs. However, the amount of tracer that is accumulated by a node depends not only on its positions in the drainage order but also on the number of lymphatic channels that enter the node and on parameters such as lymph flow rate [20]. The exact tracer patterns of a tumor site can simultaneously drain to more than one sentinel node, and differentiating a true SN from a secondary echelon node can be difficult. Goyal et al. reported that the false-negative rate in 842 clinically node-negative patients was 10.1% in those who had one SLN harvested compared to 1.1% in those with multiple SLNs (three or more) removed (*P* = 0.010) [21]. In the NSABP B-32, the FN rates were 17.7% if only one node was resected, 10% if two, 6.9% if three, 5.5% if four, and 1% if five or more [27]. These results should not translate into routine removal of multiple nodes but rather pose a question about the SLN procedure optimization. Moreover, variations in lymphatic channels may exist and thus may influence identification of positive sentinel nodes according to different procedures and modalities. To determine the effect of different injection sites for radiotracer and blue dye on FN rate the FRANSENODE trial strongly validates the periareolar injection technique giving the high detection rate (99.1%) of SLNB and high concordance (95.6%), thereby improving probe detection [22]. However, identification rate could also represent a false reassurance in the accurate analysis of the tumor lymphatic network if considering the different drainage patterns compared to the optimal injection path. According to Anan et al., no matter which mapping agents are used, a two-site injection method based on subareolar (SA) and peritumoral (PT) technique may be superior to a one-site injection in limiting the false-negative rate of SLNB for early breast cancer [23]. The success of sentinel channels mapping is optimized not only by using dye and isotope but also by a combination of PT and SA injections, which is useful for identifying the potential discordance between the hot and the blue nodes found in the 8.5% of patients by Noguchi et al. [28]. Based on available data, the intraparenchymal or peritumoral technique is necessary to evidence cases of extra-axillary drainage (internal mammary or infra- or supraclavicular) that is present in about 20% of patients and focus the discussion on several points that are still open to debate [24]. Therefore, surgical results are optimized when preoperative lymphoscintigraphy mapping is obtained in addition to preoperative probe detection. Failure to visualize a sentinel node is predictive of difficult intervention, and negative scintigraphy also heralds a higher risk of axillary involvement. In Brenot-Rossi's et al. experience, positive nodes were identified in 28.5% of patients with successful axillary drainage and in 63.3% with unsuccessful axillary drainage. More than four invaded axillary nodes (*P* < 0.0001) and the presence of lymphovascular invasion in the breast tumor (*P* = 0.004) were the only significant variables of univariate analysis, although multivariate analysis showed that only the increased number of invaded nodes was statistically significant [25]. Then, these results seem to indicate a prognostic predictive value for this event and, in cases of nonvisualization, some authors superficially reinject after peritumoral injection. However, sentinel nodes that appeared with rescue injections were associated with a significantly higher false-negative rate (23.8%) in patients for whom deep and peritumoral injections had failed [29]. Thus, when scintigraphy is negative after an adequate delay, one should check for the presence of macrometastases by ultrasound before surgery. When no sentinel node is identified at surgery, the new criteria produced by clinical trials might form the basis for designing a target node sampling according to the patient's risk of nodal involvement.

## 3. Anatomical Findings and Current Knowledge Prospects

**Figure 1:**
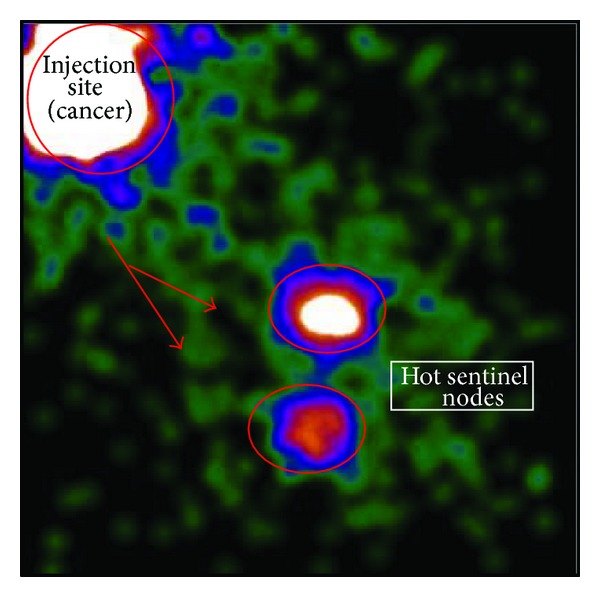
Intraoperative lymphoscintigraphy of patient with nonpalpable left breast cancer 3 hours after peritumoral injection of radiotracer (^99m^TC-labelled human albumin microcolloid). Anterior view shows 2 axillary sentinel nodes.

**Figure 2:**
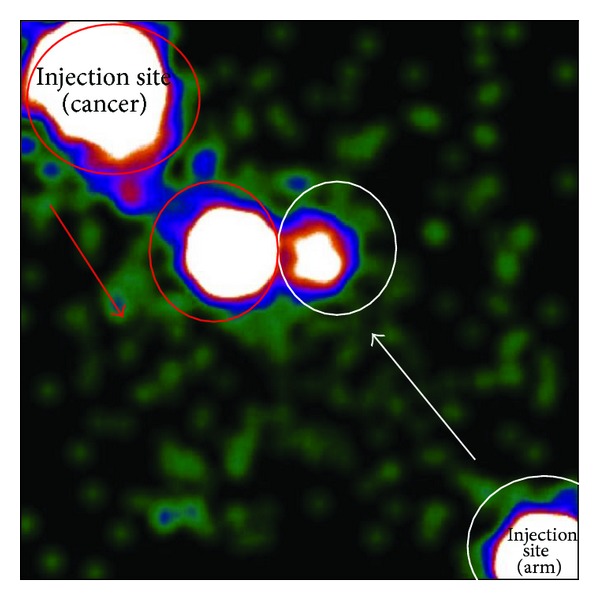
Intraoperative lymphoscintigraphy (^99m^TC-labelled human albumin microcolloid, subdermally injection) of patient with palpable left breast cancer (red circles) 1 h after previous axillary reverse mapping through a lower dose of radiotracer injected subcutaneously in the intramuscular groove of inner ipsilateral arm (white circles). Anterior view shows the progressive acquisition of two different patterns of lymphatic drainage carried out in separate times.

The exact route of breast lymphatic drainage to the axilla continues to be debated, although recent studies provide more knowledge on the anatomical network. Different drainage patterns may help to investigate some important unresolved clinical problems, including different detection rates in different studies and high false-negative rates. Reasons for this have been technical, related to personal surgeon experience or the site of the radioactive tracer, which may not reach the lymph node especially if sited in a peritumoral position. Suami et al. suggested that anatomical studies of the breast and anterior upper torso might help explain the percentages of false-negative SLNBs and identify an appropriate injection site for SLN detection. The cross sectioned specimens, radiographed to provide three dimensional images of lymph collecting vessels, showed that although the majority of the breast drains to one sentinel node, every breast area is drained by more than one first-tier node in each study [7] (Figure 1). Although the intradermal injection technique has attractive practical features, the relationship between the superficial lymphatics and the adult cancerous breast tissues has not been adequately described. There is currently insufficient certainty that drainage of tracer injected anywhere in or underneath the skin of the breast reflects drainage from the cancer, and some authors state that there is no constant route via the subareolar plexus [30]. Also, evidence that some of the torso vessels (perforating lymphatic system) pass from the periphery through the breast tissue on their way towards the axilla questioned the concrete evidence of a centripetal anatomical lymphatic pathway towards the subareolar plexus. Turner-Warwick revealed a direct pathway from the tumor injection site to the axillary lymph node, concluding that the collecting vessels through the breast contribute to the drainage pattern and lymphoscintigraphy examinations [31]. The perforating system is connected to the deep lymphatic system and these collecting vessels have the same appearance as the superficial lymphatics as they course with the internal thoracic blood vessels. The color-coded diagrams of dissection, to simulate various injection sites for lymphatic mapping and sentinel node biopsy in breast cancer, suggest a mechanism for false-negative SLNB since more than one sentinel node drains every glandular tissue area. As reviewed by Wang et al., six lymphatic drainage patterns based on three types of sentinel lymphatic channels (SLCs) were found in 107 early stage breast cancer patients. The drainage pattern was a significant risk factor for unsuccessful identification of sentinel lymph node (*P* < 0.001) and false-negative SLNB; whereas, patient age, tumor location, tumor size, pathology, and tumor grade do not affect the sentinel detection rate [6]. This helps explain the fact that in all quadrants of the breast, cancer has the potential to spread via the internal mammary lymphatics, especially if the tumor is medial or deep in the breast parenchyma. The variable contribution of perforating lymphatics along the branches of the internal mammary artery to lymphatic drainage cannot be clinically predicted, thus suggesting that accurate mapping for additional information requires peritumoral injection and alternative operating pathways in surgical management. Moreover variations in axillary clearance put the arm lymphatics at risk for disruption during axillary lymph node surgery [32]. The recent data collected on successful identification and protection of the arm lymphatics, in addition to further investigations of lymphedema occurrence, define any crossover between a hot breast node and a blue arm node. The goal of the axillary reverse mapping (ARM) pilot study was to develop a technique to identify and preserve arm lymphatic drainage, thereby decreasing the likelihood of disruption during ALND and, to a lesser degree, SLNB. In a series of 220 patients undergoing sentinel biopsy with or without axillary dissection, Boneti et al. revealed a rare ARM-SLN crossover rate (2.8%) but a more frequent anatomical juxtaposition in 40.6% of patients placing at risk for disruption during lymphadenectomy [33] (Figure 2). Disruption of the blue ARM node due to proximity to the hot SLN may also explain the high rate of lymphedema seen after SLNB. Metastasis of the arm node develops in 0–43% of patients according to several topics [34–36]. Early reports have suggested that arm lymph nodes rarely contain metastatic disease and that their preservation may translate into a lower incidence of postoperative lymphedema, especially in cases of completion ALND. Other authors have used the technique during ALND in node-positive patients and found nonnegligible prevalence (10%–20%) of disease involving ARM nodes. Bedrosian et al. reported a Phase I trial conducted in 30 patients with cytologically documented axillary metastasis, 23 (77%) of whom received preoperative therapy. Of 11 patients who had axillary metastasis and at least one ARM lymph node identified, 18% had metastasis to the ARM node. The small number of patients enrolled in this pilot trial may have resulted in an overestimate of the risk of metastasis to the ARM node. Furthermore, even if axillary metastases were noted in 60% of cases, the disease clinical stage was II (A-B) and III (A-B-C) in 36.6% and 53.3%, respectively [37]. Nos et al. reported that ARM nodes showed metastatic involvement in 3 of 21 patients with N1-3 (14%) [35]. Similarly, in a study by Noguchi et al., the metastatic rate for arm nodes was 3 of 7 patients (43%) with a clinically positive node or positive SLNB, and all were pN3, highlighting a potential contraindication for high N stage [36]. Therefore, additional prospective studies in this patient population are warranted to provide a more comprehensive understanding regarding the relation between ARM lymph nodes with oncologic and functional endpoints. It is also important to point out that the concordance between the radioactive sentinel node and the blue ARM node needs to be better defined on the basis of clinical and pathological prognostic factors, varying in clinical trials from 3.9% to 18.9% [38, 39]. If more patients are included, statistical analysis would be possible and the relationship between the location and metastasis of arm nodes would be identified. The novel findings on anatomical mapping knowledge could produce further advances in surgical techniques, thus leading to optimal information for axillary staging and lymphedema microsurgical preventive healing approaches [40].

## 4. Conclusions

There are clearly unfinished areas of research in the field of axillary lymph node surgery but the nodal status still remains the primary prognostic discriminant in breast cancer patients. The aims of new alternative diagnostic pathways were to evaluate the effects on patient outcomes and cost-effectiveness of enhanced imaging (MRI and PET) compared with standard techniques in the assessment of axillary lymph node metastases. Lymphatic mapping with sentinel node approach is one of the most interesting recent developments in surgical oncology, allowing patients with metastasis to be treated in early phase without submitting other patients to unnecessary regional dissection. The technique assumes orderly progression of tumour spread to the regional node and biopsy of the first nodes in the lymphatic chain at risk for metastasis should therefore reflect involvement of the remaining nodes. However, the lymphatic interconnections variability makes the diagnostic procedure complex and with different results. Lymphatic mapping for early breast cancer has become the standard of care but there is as yet no single study that demonstrates conclusively which particular sentinel node protocol is best for a specific patient. It may also be useful for future studies to report diagnostic accuracy according to subgroups of patients with different stages and molecular subtypes of primary breast tumors in order to inform management decisions for these categories. The false-negative rate is one of the safety parameters of SLNB and this result might impair patient outcomes for several reasons. Optimization of procedure could be implemented by dual mapping injection site skills, resection of all hot or blue nodes through tracer combination, and improvement in atypical drainage patterns mapping. Variations in lymphatic channels may exist and thus influence detection of positive sentinel nodes. Frequently, more than one sentinel node drained the breast and the risk of missed axillary disease after negative SLNB would also progressively increase depending on tumor size. This anatomical analysis suggests safety measures in patients with high probability of node involvement through a renewed interest in surgical management. The perspective of a guided axillary sampling (GAS) could represent a potential development of recent anatomical and functional acquisitions, offering a dynamic technique shared according to clinical and anatomical disease parameters. Evolutions in lymphatic drainage mapping and its interconnections could explore the concept of disease progression with a new therapeutic value for the axillary staging procedures. Furthermore, lymph node dissection may adopt a conservative approach through the evaluation of upper arm lymphatics during axillary surgery, thus defining a functional model aimed at the reduction of short- and long-term adverse events. Quality results in breast cancer surgery need to generate oncological safety devoid of complications through renewed clinical experience.

## Figures and Tables

**Table 1:** Clinical results and accuracy of non-invasive diagnostic imaging techniques (US, PET-TC, and MRI) in breast cancer assessment of axillary node metastases of studies included in systematic review.

Trial	Evaluable pts	Diagnostic technique	Sensitivity range	Specificity range	FN rate	Selected characteristics
	722	US	64.4–72.7%	44.1–97.9%	7–17.5%	Size criterion Both palpable/nonpalpable nodes
	706	US	54.7–92.3%	80.4–97.1%	4.2–17.4%	Morphologic criterion Both palpable/nonpalpable nodes
Alvarez et al., 2006 [8]	582	US	48.8–87.1%	55.6–97.3%	5.3–23.1%	Size criterion Nonpalpable nodes
	708	US	26.4–75.9%	88.4–98.1%	8.4–26.9%	Morphologic criterion Nonpalpable nodes
	822	US-needle biopsy	25.9–94.9%	96.9–100%	2.3–40.9%	Only needle-biopsied cases
Choi et al., 2012 [9]	483	US	50%	80.7%	3.7–15.5%	Pathologic N-stage classification
Cooper et al., 2011 [5]	2591	PET or PET/TC	56–66%	93–96%	7.2% 0.5%	Sampling methods replaced with PET PET added to sampling methods
Peare et al., 2010 [10]	2460	PET	20–100%	64–100%	13.7%	Staging techniques comparison
Cooper et al., 2011 [5]	307	MRI	65–98%	73–100%	1.9% 0.1%	Sampling methods replaced with MRI MRI added to sampling methods
Lu et al., 2013 [13]	32	MRL	86.2%	95.3%	9%	Enhancement defects criteria Lymphatic vessel dilation study
Kwak et al., 2013 [14]	57	US/MRI/PET	82.1%	45.9%	1.85–4.03%	Complete preoperative scanning

MRL: magnetic resonance lymphangiography.

**Table 2:** Characteristics of studies including systematic review and suggested issues (QIs) for improvement in sentinel node procedures.

Trial	SLN identified	Evaluable pts	FN rate	Tracers	Injection sites	Quality indicators
Veronesi et al., 2010 [17]	100%	516	1.2%	Radiocolloid	Close to the tumor	Wide exclusion criteria
Zavagno et al., 2008 [18]	95%	662	16.7%	Radiocolloid	Subdermally	Wide inclusion criteria
Goyal et al., 2006 [21]	96.1%	842	6.7%	Both dye and radiocolloid	Peritumoral	Dual mapping tracers
Krag et al., 2007 [27]	97.2%	2619	1–17%	Both dye and radiocolloid	Peritumoral	Dual mapping tracers/number of removed nodes
Buonomo et al., 2009 [19]	97.7%	168	3.7%	Radiocolloid	Subdermally/peritumoral	High Risk DCIS treatment Pathologic evaluation protocol
Anan et al., 2006 [23]	96.6%	145	4.9%	Dye	Subareolar/peritumoral	Dual site mapping Dual mapping tracers
Noguchi, 2009 [28]	99.5%	201	8.5%	Both dye and radiocolloid	Subareolar/peritumoral	Dual site mapping Dual mapping tracers
Brenot-Rossi 2003 [25]	90.7%	332	6.6%	Radiocolloid	Subareolar/peritumoral	Failure/negative scintigraphy Unsuccessful mapping/skipping foci
Bourgeois 2008 [29]	90%	521	5–23.8%	Radiocolloid	Subareolar/peritumoral	Unsuccessful mapping Rescue injection technique

SLN: sentinel lymph node; QIs: quality indicator.

## References

[1] West Midlans Cancer Intelligence Unit (2009). *0–10 Year Relative Survival for Cases of Breast Cancer by Stage Diagnosed in the West Midlands 1985–1989 Followed Up to the End of 1999, as at January 2002*.

[2] National Institute for Health and Clinical Excellence (NICE) (2009). *Early and Locally Advanced Breast Cancer: Diagnosis and Treatment*.

[3] Blanchard DK, Donohue JH, Reynolds C (2003). Relapse and morbidity in patients undergoing sentinel lymph node biopsy alone or with axillary dissection for breast cancer. *Archives of Surgery*.

[4] Lyman GH, Giuliano AE, Somerfield MR (2005). American Society of Clinical Oncology guideline recommendations for sentinel lymph node biopsy in early-stage breast cancer. *Journal of Clinical Oncology*.

[5] Cooper KL, Meng Y, Harnan S (2011). Positron emission tomography (PET) and magnetic resonance imaging (MRI) for the assessment of axillary lymph node metastases in early breast cancer: systematic review and economic evaluation. *Health Technology Assessment*.

[6] Wang M, Zhou W, Zhao Y (2012). A novel finding of sentinel lymphatic channels in early stage breast cancer patients: which may influence detection rate and false-negative rate of sentinel Lymph Node Biopsy. *PLoS ONE*.

[7] Suami H, Pan W-R, Mann GB, Taylor GI (2008). The lymphatic anatomy of the breast and its implications for sentinel lymph node biopsy: a human cadaver study. *Annals of Surgical Oncology*.

[8] Alvarez S, Añorbe E, Alcorta P, López F, Alonso I, Cortés J (2006). Role of sonography in the diagnosis of axillary lymph node metastases in breast cancer: a systematic review. *The American Journal of Roentgenology*.

[9] Choi JS, Kim MJ, Moon HJ, Kim EK, Yoon JH (2012). False negative results of preoperative axillary ultrasound in patients with invasive breast cancer: correlations with clinicopathologic findings. *Ultrasound in Medicine and Biology*.

[10] Peare R, Staff RT, Heys SD (2010). The use of FDG-PET in assessing axillary lymph node status in breast cancer: a systematic review and meta-analysis of the literature. *Breast Cancer Research and Treatment*.

[11] Erguvan-Dogan B, Whitman GJ, Kushwaha AC, Phelps MJ, Dempsey PJ (2006). BI-RADS-MRI: a primer. *The American Journal of Roentgenology*.

[12] Lehman CD, Schnall MD (2005). Magneitc resonance imaging. *Breast Cancer Research*.

[13] Lu Q, Hua J, Kassir MM (2013). Imaging lymphatic system in breast cancer patients with magnetic resonance lymphangiography. *PLoS ONE*.

[14] Kwak HY, Chae BJ, Bae JS (2013). Feasibility of sentinel lymph node biopsy in breast cancer patients clinically suspected of axillary lymph node metastasis on preoperative imaging. *World Journal of Surgical Oncology*.

[15] Blamey RW, Hornmark-Stenstam B, Ball G (2010). ONCOPOOL: a European database for 16,944 cases of breast cancer. *European Journal of Cancer*.

[16] Mansel RE, Fallowfield L, Kissin M (2006). Randomized multicenter trial of sentinel node biopsy versus standard axillary treatment in operable breast cancer: the ALMANAC trial. *Journal of the National Cancer Institute*.

[17] Veronesi U, Viale G, Paganelli G (2010). Sentinel lymph node biopsy in breast cancer: ten-year results: of a randomized controlled study. *Annals of Surgery*.

[18] Zavagno G, De Salvo GL, Scalco G (2008). A randomized clinical trial on sentinel lymph node biopsy versus axillary lymph node dissection in breast cancer: results of the sentinella/GIVOM trial. *Annals of Surgery*.

[19] Buonomo OC, Orsaria P, Contino G (2009). Pathological classification of DCIS and planning of therapeutic management. *Anticancer Research*.

[20] Nieweg OE, Tanis PJ, Kroon BBR (2001). The definition of a sentinel node. *Annals of Surgical Oncology*.

[21] Goyal A, Newcombe RG, Chhabra A, Mansel RE (2006). Factors affecting failed localisation and false-negative rates of sentinel node biopsy in breast cancer: results of the ALMANAC validation phase. *Breast Cancer Research and Treatment*.

[22] Rodier J-F, Velten M, Wilt M (2007). Prospective multicentric randomized study comparing periareolar and peritumoral injection of radiotracer and blue dye for the detection of sentinel lymph node in breast sparing procedures: FRANSENODE trial. *Journal of Clinical Oncology*.

[23] Anan K, Mitsuyama S, Kuga H (2006). Double mapping with subareolar blue dye and peritumoral green dye injections decreases the false-negative rate of dye-only sentinel node biopsy for early breast cancer: 2-site injection is more accurate than 1-site injection. *Surgery*.

[24] Hindié E, Groheux D, Espie M (2009). Sentinel node biopsy in breast cancer. *Bulletin du Cancer*.

[25] Brenot-Rossi I, Houvenaeghel G, Jacquemier J (2003). Nonvisualization of axillary sentinel node during lymphoscintigraphy: is there a pathologic significance in breast cancer?. *Journal of Nuclear Medicine*.

[26] Hindié E, Groheux D, Brenot-Rossi I, Rubello D, Moretti J-L, Espié M (2011). The sentinel node procedure in breast cancer: nuclear medicine as the starting point. *Journal of Nuclear Medicine*.

[27] Krag DN, Anderson SJ, Julian TB (2007). Technical outcomes of sentinel-lymph-node resection and conventional axillary-lymph-node dissection in patients with clinically node-negative breast cancer: results from the NSABP B-32 randomised phase III trial. *The Lancet Oncology*.

[28] Noguchi M, Inokuchi M, Zen Y (2009). Complement of peritumoral and subareolar injection in breast cancer sentinel lymph node biopsy. *Journal of Surgical Oncology*.

[29] Bourgeois P, Nogaret JM, Veys I (2008). Isotope labelling and axillary node harvesting strategies for breast cancer. *European Journal of Surgical Oncology*.

[30] Tanis PJ, Nieweg OE, Valdés Olmos RA, Kroon BBR (2001). Anatomy and physiology of lymphatic drainage of the breast from the perspective of sentinel node biopsy. *Journal of the American College of Surgeons*.

[31] Turner-Warwick RT (1959). The lymphatics of the breast. *The British journal of surgery*.

[32] Thompson M, Korourian S, Henry-Tillman R (2007). Axillary reverse mapping (ARM): a new concept to identify and enhance lymphatic preservation. *Annals of Surgical Oncology*.

[33] Boneti C, Korourian S, Diaz Z (2009). Scientific impact award: axillary reverse mapping (ARM) to identify and protect lymphatics draining the arm during axillary lymphadenectomy. *The American Journal of Surgery*.

[34] Jung EC, Young SJ, Su HK, Soo JL (2009). Preservation of lymphatic drainage of arm during axillary procedure in breast cancer patients. *Journal of Breast Cancer*.

[35] Nos C, Kaufmann G, Clough KB (2008). Combined axillary reverse mapping (ARM) technique for breast cancer patients requiring axillary dissection. *Annals of Surgical Oncology*.

[36] Noguchi M, Yokoi M, Nakano Y (2010). Axillary reverse mapping with indocyanine fluorescence imaging in patients with breast cancer. *Journal of Surgical Oncology*.

[37] Bedrosian I, Babiera GV, Mittendorf EA (2010). A phase I study to assess the feasibility and oncologic safety of axillary reverse mapping in breast cancer patients. *Cancer*.

[38] Boneti C, Korourian S, Bland K (2008). Axillary reverse mapping: mapping and preserving arm lymphatics may be important in preventing lymphedema during sentinel lymph node biopsy. *Journal of the American College of Surgeons*.

[39] Kang Choi J S, Jeon Y, Lee S, Bae Y (2009). Preservation of lymphatic drainage from arm in breast cancer surgery: is it safe?. *Cancer Research*.

[40] Boccardo F, Casabona F, De Cian F (2009). Lymphedema microsurgical preventive healing approach: a new technique for primary prevention of arm lymphedema after mastectomy. *Annals of Surgical Oncology*.

